# Efficacy of Proprioceptive Training on Plantar Pressure and Jump Performance in Volleyball Players: A Proof-of-Principle Study

**DOI:** 10.3390/s23041906

**Published:** 2023-02-08

**Authors:** Nicola Marotta, Lucrezia Moggio, Dario Calafiore, Emanuele Prestifilippo, Riccardo Spanó, Anna Tasselli, Vera Drago Ferrante, Marco Invernizzi, Alessandro de Sire, Antonio Ammendolia

**Affiliations:** 1 Department of Medical and Surgical Sciences, University of Catanzaro “Magna Graecia”, 88100 Catanzaro, Italy; 2 Rehabilitation Unit, Ospedale degli Infermi, 13875 Biella, Italy; 3 Physical Medicine and Rehabilitation Unit, Department of Neurosciences, ASST Carlo Poma, 46100 Mantova, Italy; 4 Physical and Rehabilitative Medicine, Department of Health Sciences, University of Eastern Piedmont “A. Avogadro”, 28100 Novara, Italy; 5 Translational Medicine, Dipartimento Attività Integrate Ricerca e Innovazione (DAIRI), Azienda Ospedaliera SS. Antonio e Biagio e Cesare Arrigo, 15121 Alessandria, Italy

**Keywords:** volleyball, mat-based, baropodometric analysis, inertial sensor, proprioception, performance, rehabilitation

## Abstract

Volleyball players are often subject to micro-traumatisms of the heel fat pad and ankle injuries. Recently, mat-based proprioceptive training has assumed a key role in recovery from these disorders. Therefore, this proof-of-principle study aimed to assess the efficacy of proprioceptive mat training on plantar pressures and athletic performance in volleyball players. The participants included adult semi-professional volleyball players allocated into two groups: an experimental group, with mat-based proprioceptive and balance training, and a control group, with a sham protocol. For the outcome, we evaluated the barefoot plantar pressure, performing an analysis on a baropodometric resistive platform. The countermovement jump and squat jump were measured using an inertial measurement unit. Nineteen subjects were included in the two groups: the active proprioceptive group (*n* = 10) or the control group (*n* = 9). The results show a more uniform redistribution of loads with pressure hindfoot relief in the experimental group compared to the control group (*p* = 0.021, RBC = 0.67). Moreover, we observed a significant increase in peak landing force and high concentric power development in the experimental group compared to the controls. Focused proprioceptive management provided hindfoot load attenuation by stimulating higher peaks of concentric force in the experimental group compared to the sham group. Even though the study included a small sample, the results obtained in this proof-of-principle study suggest a positive role of proprioceptive stimulation in the inter-seasonal scenario for volleyball players to improve their jump performance and reduce the micro-traumatisms of the heel fat pad and the ankle injury rate. However, further studies performed on larger samples are needed to confirm these preliminary results.

## 1. Introduction

Volleyball is one of the most popular sports, with 500 million players worldwide, due to its broad age-appropriate accessibility, minimal equipment and cost requirements, and the ability to play indoors and outdoors [1,2]. The sport implicates constant, prompt lateral action in combination with complex ballistic motions in reaction to outer stimuli [3]. Since the 1980s, there has been a conspicuous gain in the number of players at different levels of experience, consequently increasing the incidence of injuries. Indeed, the lower extremities are most commonly injured, involving repetitive athletic jumping gestures, either of acute or overuse nature, despite the non-contact essence of volleyball [4,5,6,7,8,9,10].

Despite several studies on the risk of injury in athletes focused exclusively on large joints (e.g., knees and ankles) [11,12,13,14], there is still poor evidence on plantar pressure modifications and fat pad atrophy in volleyball players [11]. The plantar fat pad of the foot is a thick layer of connective tissue that runs underneath the foot, uniquely specialized to permit discomfort-free weight-bearing during daily activities and provide a cushioning system to reduce the effects of pressure, friction, and load forces [15]. Plantar fat pad thickness adaptation or anterior shift can act as a key factor in the development of metatarsalgia, which is one of the most common foot disorders [16,17]. 

Metatarsal heads with an increase in plantar loads might underlie a fat pad thinning. Indeed, a fat pad alteration might enhance the metatarsal loads, and this pressure increase could lead to undesirable plantar keratoses and other frequent disorders related to metatarsalgia [18]. Moreover, biomechanics adaptations can be due to changes in foot plantar pressure, that is, an increased load on the medial arch is usually suggestive of foot pronation shifting the sagittal plane kinematics, inclining volleyball players to injuries [19]. At the same time, changes in the foot plantar pressure may also cause changes in the foot biomechanics. A pronated foot might be affected by fatigue development, increasing the athlete’s predisposition to an injury [20,21]. Regarding proprioception, there is increasing focus on prevention and injury management, especially in the field of sports. Proprioception refers to awareness of the body and its limbs and their location as they move through space. Thus, proprioceptive mechanisms prove crucial in balance control through continuous neuromuscular feedback [22,23]. Recently, proprioceptive conditioning has assumed a key role in physical training [24,25,26]; however, to date, few studies have investigated the role of proprioception training and its impact on plantar foot biomechanics in volleyball athletes.

Preventive exercises and rehabilitation interventions can protect and strengthen the ankle and foot region from injuries [21,27,28,29]. These protocols may include joint stability exercises, balance training, proprioceptive training, plyometric exercises, and specific skill training [30]. Recently, devices to train proprioception and evaluate plantar pressure have been shown to play a role in physical performance conditioning [31,32,33,34,35,36]; however, to date, the impact of proprioceptive mat training has not been assessed yet. 

Therefore, the present study aimed to examine the effects of proprioceptive conditioning performed on a specific proprioceptive device on plantar pressure and jump performance in a small sample of volleyball players.

## 2. Materials and Methods

### 2.1. Participants

This proof-of-principle sham-controlled study was conducted at the Rehabilitation Unit of the University Hospital “Mater Domini” of Catanzaro, Italy, and approved by the Institutional Review Board (number: 115/2022). This study was conducted according to the Ethical Principles for Medical Research Involving Human Subjects delineated in the Declaration of Helsinki. All subjects were fully informed about all interventions and outcomes and signed a written informed consent form before participation.

The inclusion criteria were: (a) adult males; (b) at least 5 years of volleyball experience; (c) no injuries in the last 12 weeks; (d) no anti-inflammatory therapy in the past 2 weeks. We excluded participants with active plantar infections or disorders or pain when performing athletic gestures and those who followed any additional lower limb-strengthening programs.

### 2.2. Intervention

After assessing the eligibility, all participants were divided through a randomization scheme with a 1:1 ratio of allocation into two groups: the experimental group, undergoing a mat-based proprioceptive and balance training, and the control group, undergoing a sham mat-based proprioceptive and balance training. In more detail, both groups performed a structured protocol in a daily session with active or sham proprioceptive intervention for 5 days a week for two weeks (10 sessions in total). 

Four Synergy Mats^®^ (Human Tecar, Unibell International, Calco (LC), Italy) of different patterns and surfaces were utilized. The platforms used were: (1) Foam Mat: Flat surface with medium density elastic response; ideal for movements with a reduced joint load; dimensions: 200 × 54 × 7 cm. (2) Dune Mat: An irregular surface that simulates sandy soil, stimulating the microcirculation and the mechanoreceptors of the foot. (3) Cobblestones Mat: Sandy soil irregular surface, stimulating the baroreceptors and activating the stabilization muscles and plantar microcirculation; dimensions: 200 × 54 × 7 cm. (4) 4-Density Mat: A combined mat consisting of a rigid frame tray with cubes of different densities and textures; dimensions: 200 × 54 × 14 cm [37].

Both groups accomplished a warm-up session with a free gait for 3 min to become familiar with the mats. In more detail, the experimental group was instructed to perform: a tandem gait, performed two-sided on the 4 surfaces for 3 min; walking lunges, performed two-sided on the 4 surfaces for 3 min with therapist disturbances; calf-raise gait, performed two-sided on the 4 surfaces for 2 min; monopodial stabilization, with stationary single stance support on 4 different surfaces for a period of at least thirty or forty seconds and with the therapist who performs small perturbations; quarter-movement pistol squat, with 3 sets of 8-10 reps per leg, on 4 surfaces; countermovement jump from unstable platforms on the different 4 surfaces (1 set of 10 jumps for each mat); athletic gesture—monopodial support on 4 different surfaces with the performance of a volley athletic gesture (4 sets of 2 min).

**Figure 1:**
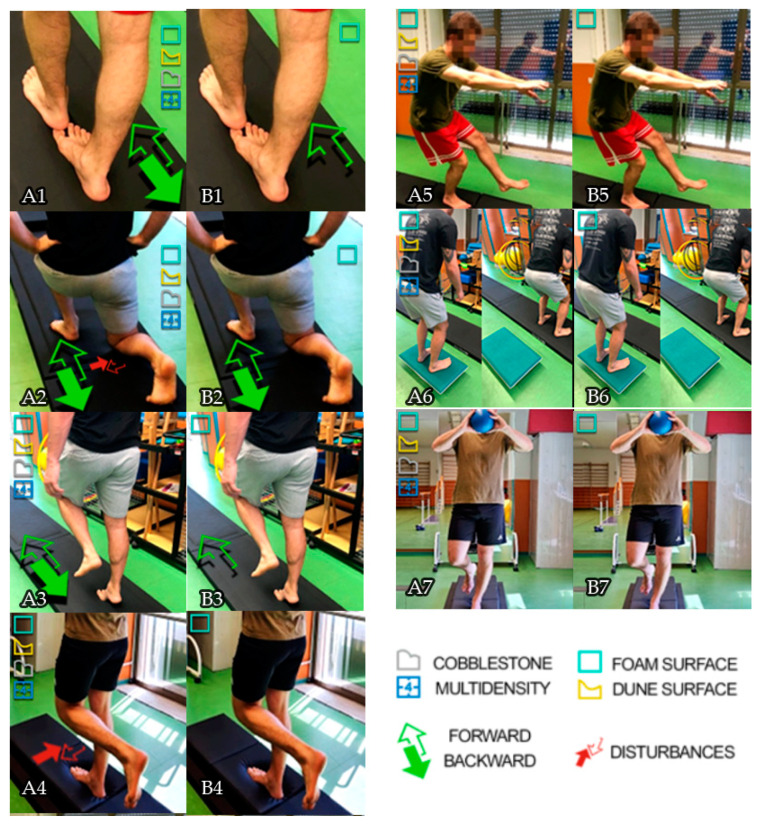
Mat proprioceptive protocol. (**A1**) Tandem gait, performed two-sided on the 4 surfaces for 3 min. (**A2**) Walking lunges, performed two-sided on the 4 surfaces for 3 min with therapist disturbances. (**A3**) Calf-raise gait, performed two-sided on the 4 surfaces for 2 min. (**A4**) Monopodial stabilization, with stationary single stance support on 4 different surfaces for a period of at least thirty or forty seconds and with the therapist who performed small perturbations. (**A5**) Quarter-movement pistol squat, with 3 sets of 8–10 reps per leg on 4 surfaces. (**A6**) Countermovement jump from unstable platforms on the different 4 surfaces (1 set of 10 jumps for each mat). (**A7**) athletic gesture—monopodial support on 4 different surfaces with the performance of a volley athletic gesture (4 sets of 2 min). (**B1**) Tandem gait, exclusively forward for 1 min. (**B2**) Walking lunges performed only forward on the foam surface for 1 min. (**B3**) Calf-raise gait performed only forward on the foam surface for 2 min. (**B4**) Monopodial stabilization, subject stationary on single stance support surface for a period of at least thirty or forty seconds. (**B5**) Quarter-movement pistol squat, 3 sets of 8–10 reps per leg on foam surface. (**B6**) Countermovement jump (CMJ) from unstable platforms on foam surface; one set of 10 jumps on the foam mat. (**B7**) Athletic gesture—monopodial support on foam surface with the performance of a volley athletic gesture (1 set of 2 min).

The control group performed exercises on a single surface for a shorter time and without stimuli from the therapist (for more details, see Figure 1). 

### 2.3. Outcome Measures

All subjects included in the study were asked to maintain a still position on a 48 cm^2^ baropodometric resistive platform (EPS R-1 model, Loran Engineering, Castel Maggiore (BO), Italy) with 2224 sensors, a pressure-array capacity of 50–350 kilo-Pascals (kPa), and a 50 Hz data-collection rate. The players were barefoot, with their feet spread apart in correspondence with the projection of their upper limbs, with the latter lying along the sides of their trunk.

Biomech Studio software (Loran Engineering, Castel Maggiore (BO), Italy) elaborated the data on the plantar pressures and center-of-pressure (COP) translations. Assessments were conducted during 3 trials of 1 min each [38,39].

The plantar surface of the foot was outlined using the same Biomech Studio software in ten areas (forefoot: A1—zone of the hallux, A2–5—zone between the second and fifth toes, M1, M2, M3, M4, and M5 zones—zones of the first, second, third, fourth, and fifth metatarsal heads, respectively; midfoot: MF areas; hindfoot: LH—lateral heel zone, MH—medial heel zone). The data were defined as the pressure loads in kPa, computed as the mean (P_mean_) and maximum (P_max_) pressures acquired from the three time series [40].

Thus, we assessed the performance tests using an inertial measurement unit (IMU, BTS G-Sensor 2, BTS Bioengineering, Milano, Italy). During the analysis, the IMU was placed in a waistband strapped tightly to the subject’s trunk. The IMU was aligned with the middle of the lumbar spine. The 70 × 40 × 18 mm IMU weighed 37 g and was able to act as a triaxial accelerometer, gyroscope, and magnetometer. The signals were collected at 100 Hz via Bluetooth^®^ connection, and the data collected were defined according to the phase in which they occurred, countermovement, propulsive, or landing phase. With the Squat Jump (SJ), the subjects began the test in an upright position, with the feet positioned in correspondence with the projection of the shoulder girdle, keeping the hands on the hips. Once the assessor gave a verbal cue to jump, the subject performed a squat by bending their knees 90° keeping this position for 1 s. From this position of a static squat, the player made a vertical jump without any countermovement. Regarding the CMJ, subjects began the testing motion still in an upright position with their hands on their hips and with an agreed signal, reaching a new squat position, but in countermovement, i.e., performing a vertical jump at maximum effort. The participants performed three maximal efforts, separated by approximately 60 s of standing recovery; the mean of the 3 jumps was used for analysis. We evaluated the following outcomes: (1) jump height (flight height plus the difference between the standing height and the takeoff height; (2) low force (lowest force during the initiation of the countermovement; (3) peak landing force or impact force, defined as the peak force occurring after ground contact when landing from the jump; (4) maximum power (calculated from the product of the force and velocity data); take-off speed (m/s) [41,42,43].

### 2.4. Statistical Analysis

All outcome measures were conducted at baseline (T0) and the end of the intervention (T1). Quantitative variables were defined by the mean ± standard deviation (SD). Yazici et al. reported that the G-walk is a reliable device for gait and jump performance data evaluation in healthy adults (all measurements had high test–retest reliability with an ICC of 0.90–0.97) [41]. The distribution of normality was proven by the Shapiro–Wilk test. To examine the result of the interventions on plantar pressure and jump data, the Wilcoxon test was verified for paired samples. To compare unpaired groups, we adopted the Mann–Whitney U test. The significance level was set at 5% (*p* ≤ 0.05) and the data were evaluated using R 4.0.5 (R foundation, Vienna, Austria).

## 3. Results

Out of the 21 athletes considered for eligibility, two were excluded (they did not meet the inclusion criterion for the occurrence of a lower limb injury). Thus, 19 subjects were included in the study and randomly allocated into two groups: the experimental (active proprioceptive protocol) group (*n* = 10) or the control group (sham proprioceptive protocol) (*n* = 9).

There were no statistically significant differences between the groups in terms of the demographic or functional features at baseline (see Table 1 for further details).

Regarding the performance of SJ, we observed a significant increase in the jump height (*p* = 0.04) and maximum strength development (*p* = 0.03) only in the experimental group. We also evaluated a significant mean speed concentric phase difference at the end of the intervention (T1) in the comparison between the two groups (*p* = 0.01).

Furthermore, in the CMJ performance analysis, we observed a significant increase in the peak landing force (*p* = 0.04) only in the experimental group. Lastly, we observed a significant difference in the maximum power at the end of the intervention in the comparison between the two groups (*p* = 0.01), as depicted in Table 2.

In the assessment of the plantar pressure, we observed a significant increase in the loads on the M2, M3, and MH zones in the control group. In contrast, regarding the experimental group, we observed a decrease in pressure at the level of the M4 zone (*p* = 0.04). Lastly, differences between the two groups were found to be significant, especially in the mid-posterior (MF, *p* = 0.02), medial (MH, *p* = 0.01), and lateral (LH, *p* = 0.01) calcaneal regions (see Table 3 for further details).

**Figure 2:**
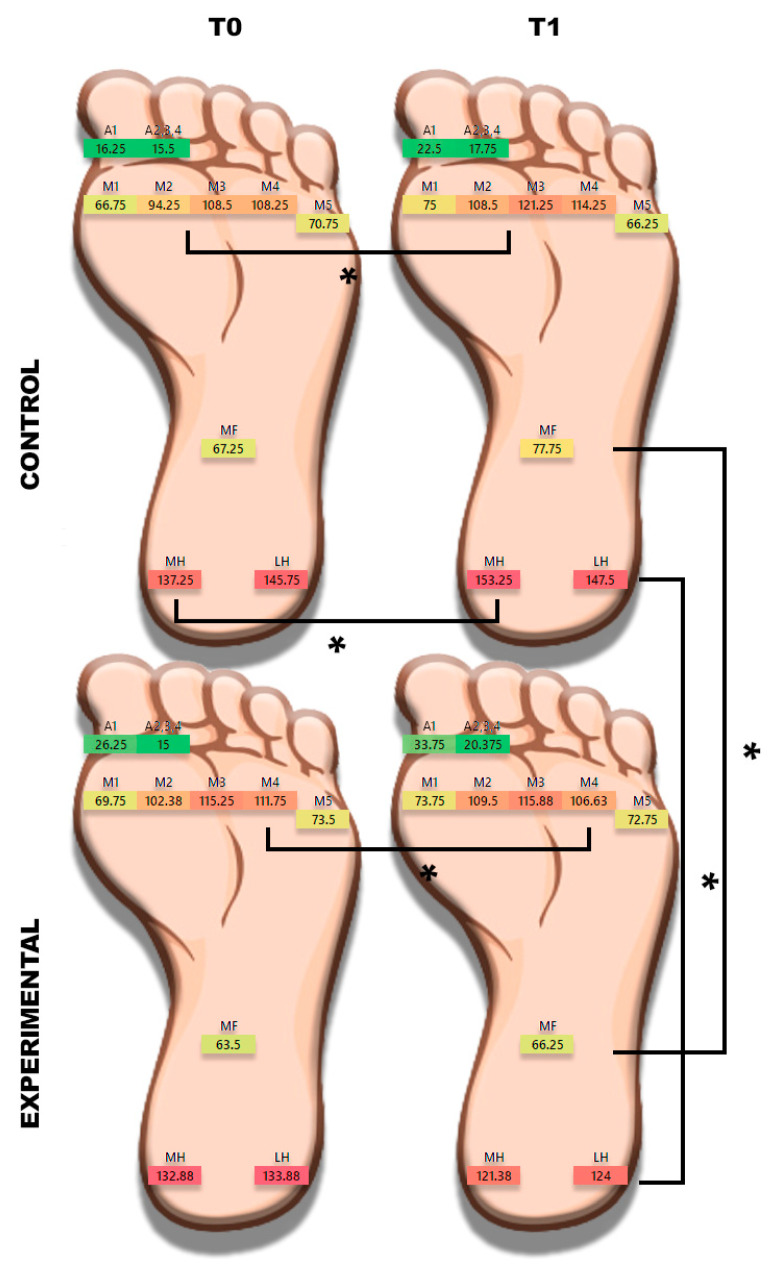
Pressure distribution 10-zone plot. * = significant difference (*p* < 0.05). Abbreviations: A1—area of the hallux; A2–5—area between the second and fifth metatarsal heads; M1, M2, M3, M4, and M5 areas—zone of the first, second, third, fourth, and fifth metatarsal heads, respectively; midfoot: MF—midfoot area; hindfoot: LH—lateral heel zone and MH—medial heel zone). Data are described as pressure loads in kPa, quantified as mean (P_mean_) pressures.

Figure 2 shows the mean pressure distributions in the two groups at each timepoint. Curiously, the control group showed a significant gain in posterior loads, while in the experimental group, the intervention appears to have provided a more uniform redistribution of pressure with hindfoot relief compared to the control group.

## 4. Discussion

This proof-of-principle study aimed to assess the efficacy of proprioceptive mat training on plantar pressures and athletic performance in volleyball athletes. To the best of our knowledge, although biomechanical and plantar pressure changes have been previously investigated, this is the first study assessing the effects of tailored proprioceptive training on plantar pressure and biomechanics in volleyball players. 

Regarding plantar pressures, the differences between the two groups were found to be significant, especially in the mid-posterior (MF, *p* = 0.02), medial (MH, *p* = 0.01), and lateral (LH, *p* = 0.01) calcaneal region. Thus, the results show a more uniform redistribution of loads with pressure hindfoot relief in the experimental group compared to the control group. Moreover, with the performance evaluation, we observed a significant increase in the peak landing force only in the experimental group and high concentric power development at the end of the intervention in the group comparison.

Volleyball is a sport that involves repeated athletic gestures throughout the athlete’s career, including spiking and blocking, which involve continuous rebound motions and dynamic biomechanical response, affecting the foot and the athlete’s plantar sole [44]. Garcia et al. [45] reported that during a single-leg drop jump, appropriate foot landing motions, such as forefoot landings, appeared to decrease the peak vertical reaction load compared to flatfoot landings. Nevertheless, in professional athletes, with their physical characteristics developing over 10–15 year periods, these movements are performed more frequently in training sessions and daily games [44]. The contact between the foot and the ground has the effect of mechanical stress on the morpho-functional ankle joint system and is the basis for the balance and support of the whole body and the projection of the center of gravity for body posture as the plantar–ground interface [45].

In terms of somatosensory aspects, the correct plantar pressure sensation is crucial for the effective functioning of the somatosensory senses [44,45,46]. Indeed, biomechanical changes in the foot can alter the plantar load distribution and consequently the foot biomechanics and spinal posture, that could be also influenced by splints [45,46,47]. Thus, an excessive load on the medial arch could underlie greater pronation of the foot, resulting from an overuse and fatigue process, generating altered kinematics of the sagittal plane, and leading the player to a greater predisposition for injury [19]. In this scenario, shoe inserts were revealed to be active in plantar heel pain management [48]. The mechanism by which they reach their influence is still basically undefined, but it might consist of a decrease in plantar loads under the heel [49,50]. This could legitimize our results because plantar proprioceptive training might hamper heel pad stiffness and reduce the energy dissipation of loads due to chronic micro-traumatisms, long volleyball careers, and aging itself [51,52].

IMU vertical jump assessments have been validated already [41,53,54], and an additional study examined the concomitant validity of IMUs associated with force plates for CMJ landing frame time analysis in professional male soccer players, reporting high ICC scores [55]. In fact, in a clinical scenario, a simple IMU is useful as it is a low-cost and transportable device compared to an optoelectronic system and/or force platforms. Both CMJ and SJ are commonly performed to estimate the aptitude to promptly develop strength during dynamic activities [56]. It is assumed that the CMJ delivers an assessment of the competence to quickly provide force in stretch-shortening cycle actions, though the SJ offers an evaluation of the ability to develop force solely during a purely concentric movement [57]. In this regard, the major findings report a significant increase in the peak landing force and a significantly higher maximum power at the end of the intervention in the group comparison during the CMJ evaluation. Parallelly, we demonstrated a significant mean concentric speed difference at the end of the intervention (T1) in the group comparison during the SJ. It is necessary to underline that training focused on proprioceptive management provided hindfoot load attenuation by stimulating higher peaks of concentric force in the experimental group compared to the sham group.

In fact, beyond a more uniform distribution of the plantar loads, relief of the rear pressures of the foot seemed to favor a greater acceptance of the counter-response and the development of greater strength in the countermovement jump. This concept could parallel the results reported in the squat jump, as a less loaded rear foot can more easily develop concentric force.

The results of this study must be viewed considering the following limitations. First, there was a small sample size, although it should be noted that the study design exploited a proof-of-principle model and, to the best of our knowledge, this is the first manuscript investigating the role of a proprioceptive mat training protocol. Second, unfortunately, it was not possible to consider a follow-up, given the difficulties of an in-season approach and the probable lack of relevance of a post-season re-evaluation. Third, the immediate effects of this proprioceptive approach on plantar pressures were analyzed; therefore, the results may not reflect long-term changes, but it could be difficult to consider external confounding factors in the evaluations. Furthermore, beyond the IMU performance evaluation, we did not thoroughly investigate the kinematics of the lower limb joints, whereas a simple and accessible sensor might guarantee more reproducibility. Lastly, an association with vertical heel pad pressure has not been fully defined and established; however, the parallel increases in performance could shed light on the influence of plantar load distribution.

## 5. Conclusions

Taken together, the findings of this proof-of-principle study suggest that mat-based proprioceptive training provides plantar pressure redistribution in volleyball players in a pre-season context. Furthermore, the mat-based proprioceptive training increased different sport performance parameters in these athletes.

Volleyball athletes are often subject to micro-traumatisms of the heel fat pad and ankle injuries. Thus, proprioceptive stimulation performed in the inter-seasonal period could be a safe and effective intervention to be added to the comprehensive athletic training of these subjects to improve their sports performance and reduce foot and ankle injury rates.

## Figures and Tables

**Table 1:** Baseline characteristics of the experimental group and control group.

	Experimental Group (*n* = 10)	Control Group (*n* = 9)	*p* Value
Age (years)	20.9 ± 6.4	19.5 ± 6.8	0.246
Height (cm)	178.8 ± 7.5	177.3 ± 6.9	0.482
Body Mass (kg)	76.2 ± 3.6	77.2 ± 4.7	0.659
Exercise Experience (years)	5.4 ± 2.3	6.6 ± 1.7	0.438

Continuous variables and parametric data are expressed as means ± standard deviations.

**Table 2:** Group descriptive performance outcomes for squat and countermovement jump.

SJ	T0	Mean	SD	RBC	T1	Mean	SD	RBC	∆T0–T1 *p* Value
Height (cm)	Experimental	28.69	1.46		Experimental	31.42	4.9		**0.04**
	Control	32.05	4.65		Control	31.05	4.0		0.14
	Between-group *p* value	0.46		−0.2	Between-group *p* value	0.88		−0.1	
Low force (kN)	Experimental	1.05	0.2		Experimental	1.44	0.5		0.06
	Control	1.04	0.08		Control	1.04	0.1		0.14
	Between-group *p* value	0.26		−0.33	Between-group *p* value	0.26		0.33	
Peak landing force (kN)	Experimental	1.68	0.39		Experimental	1.54	0.4		0.06
	Control	2.09	0.66		Control	2.09	0.6		1
	Between-group *p* value	0.26		−0.3	Between-group *p* value	0.13		−0.4	
Max power (kW)	Experimental	3.79	0.66		Experimental	4.49	1.2		**0.03**
	Control	4.13	0.06		Control	3.93	0.1		0.56
	Between-group *p* value	0.06		−0.6	Between-group *p* value	0.46		−0.2	
Mean speed concentric phase (m/s)	Experimental	1.14	0.28		Experimental	1.44	0.1		0.06
	Control	1.33	0.01		Control	1.34	0.0		1
	Between-group *p* value	0.73		−0.1	Between-group *p* value	**0.01**		**0.78**	
Peak speed (m/s)	Experimental	2.61	0.11		Experimental	2.81	0.4		0.06
	Control	2.78	0.12		Control	2.98	0.1		0.05
	Between-group *p* value	**0.01**		**−0.8**	Between-group *p* value	0.73		−0.1	
Take-off speed (m/s)	Experimental	2.69	0.13		Experimental	2.72	0.4		0.23
	Control	2.53	0.13		Control	2.69	0.1		**0.04**
	Between-group *p* value	0.01		0.72	Between-group *p* value	0.46		−0.2	
**CMJ**	**T0**	**Mean**	**SD**	**RBC**	**T1**	**Mean**	**SD**	**RBC**	**∆T0-T1 *p* value**
Height (cm)	Experimental	34.09	4.04		Experimental	34.6	5.2		0.61
	Control	34.85	3.8		Control	34.85	3.8		1
	Between-group *p* value	0.46		−0.2	Between-group *p* value	0.73		0.11	
Low force (kN)	Experimental	2.04	0.4		Experimental	1.59	0.5		0.04
	Control	0.85	0.1		Control	0.79	0.2		0.65
	Between-group *p* value	**0.01**		**1**	Between-group *p* value	**0.01**		**1**	
Peak landing force (kN)	Experimental	1.77	0.13		Experimental	1.44	0.3		**0.04**
	Control	1.64	0.24		Control	1.59	0.1		0.22
	Between-group *p* value	0.46		0.22	Between-group *p* value	0.26		−0.33	
Max power (kW)	Experimental	5.08	1.64		Experimental	5.39	1.3		0.59
	Control	4.28	0.18		Control	4.31	0.1		0.72
	Between-group *p* value	0.06		0.56	Between-group *p* value	**0.01**		**1**	
Mean speed concentric phase (m/s)	Experimental	1.04	0.15		Experimental	1.34	0.3		0.05
	Control	1.59	0.05		Control	1.49	0.2		0.65
	Between-group *p* value	**0.01**		**−1**	Between-group *p* value	0.06		−0.56	
Peak speed (m/s)	Experimental	2.95	0.2		Experimental	2.98	0.3		0.67
	Control	2.96	0.14		Control	2.96	0.1		0.71
	Between-group *p* value	0.73		0.11	Between-group *p* value	0.73		0.11	
Take-off speed (m/s)	Experimental	2.89	0.21		Experimental	2.91	0.3		0.34
	Control	2.89	0.14		Control	2.99	0.3		0.21
	Between-group *p* value	0.73		0.11	Between-group *p* value	0.73		0.11	

Continuous variables and parametric data are expressed as means ± standard deviations. Abbreviations: CMJ: countermovement jump, SJ: squat jump. RBC: rank biserial correlation.

**Table 3:** Group descriptive plantar pressures.

	T0	Mean	SD	RBC	T1	Mean	SD	RBC	*∆T0–T1 p Value*
	Experimental	25.11	8.33		Experimental	32.11	9.99		*0.07*
A1	Control	16.25	11.2		Control	22.5	14.2		*0.08*
	*Between-group p value*	0.13		*−0.44*	*Between-group p value*	0.09		*−0.5*	
	Experimental	15.11	10.9		Experimental	20.22	10.4		*0.06*
A2–A4	Control	15.5	9.7		Control	17.75	11.1		*0.14*
	*Between-group p value*	0.47		*0.22*	*Between-group p value*	0.96		*0.03*	
	Experimental	68.56	9.75		Experimental	72.89	10.1		*0.65*
M1	Control	66.75	6.69		Control	75	8.72		*0.06*
	*Between-group p value*	0.73		*0.11*	*Between-group p value*	0.73		*0.11*	
	Experimental	102.1	12		Experimental	108.8	12.6		*0.56*
M2	Control	94.25	4.3		Control	108.5	7.95		***0.03***
	*Between-group p value*	0.11		*−0.47*	*Between-group p value*	0.81		*0.08*	
	Experimental	116.1	15.9		Experimental	114.4	12.7		*0.76*
M3	Control	108.5	4.44		Control	121.3	8.4		***0.03***
	*Between-group p value*	0.27		*−0.33*	*Between-group p value*	0.31		*0.31*	
	Experimental	112.7	10.4		Experimental	105.1	9.48		***0.04***
M4	Control	108.3	15.1		Control	114.3	13.5		*0.06*
	*Between-group p value*	0.47		*−0.22*	*Between-group p value*	0.13		*0.44*	
	Experimental	73.33	5.05		Experimental	70.56	12.7		*0.14*
M5	Control	70.75	12.6		Control	66.25	10.5		*0.05*
	*Between-group p value*	0.53		*−0.19*	*Between-group p value*	0.88		*−0.06*	
	Experimental	65	14.4		Experimental	65.11	14.5		*0.14*
MF	Control	67.25	6.25		Control	77.75	4.23		*0.06*
	*Between-group p value*	0.88		*−0.06*	*Between-group p value*	**0.02**		***0.67***	
	Experimental	132.1	20.5		Experimental	121.1	18.6		*0.74*
MH	Control	137.3	3.15		Control	153.3	17.6		***0.04***
	*Between-group p value*	0.33		*0.32*	*Between-group p value*	**0.01**		***0.75***	
	Experimental	135.2	15.3		Experimental	123.9	14.3		*0.05*
LH	Control	145.8	2.55		Control	147.5	9.02		*0.12*
	*Between-group p value*	0.16		*0.42*	*Between-group p value*	***0.01***		***0.89***	

Continuous variables and parametric data are expressed as means ± standard deviations. The data are described as pressure loads in kPa, quantified as mean (Pmean) pressures. Abbreviations= A1—zone of the first toe, hallux; A2-5—zone between the second and fifth toes; M1, M2, M3, M4, and M5 zones—zone of the first, second, third, fourth, and fifth metatarsal heads, respectively; midfoot: MF–—midfoot zone; hindfoot: LH—lateral heel zone and MH—medial heel zone). RBC: rank biserial correlation.

## Data Availability

Not applicable.
